# Plausible Role of Stem Cell Types for Treating and Understanding the Pathophysiology of Depression

**DOI:** 10.3390/pharmaceutics15030814

**Published:** 2023-03-02

**Authors:** Punya Sachdeva, Seongmin Ji, Shampa Ghosh, Soumya Ghosh, Manchala Raghunath, Hyunjin Kim, Rakesh Bhaskar, Jitendra Kumar Sinha, Sung Soo Han

**Affiliations:** 1 GloNeuro, Sector 107, Vishwakarma Road, Noida 201301, India; 2 School of Chemical Engineering, Yeungnam University, Gyeonsang 38541, Republic of Korea; 3 ICMR—National Institute of Nutrition, Tarnaka, Hyderabad 500007, India; 4 Research Institute of Cell Culture, Yeungnam University, 280 Daehak-Ro, Gyeongsan 38541, Republic of Korea

**Keywords:** depression, stem cells, molecular pathways, neurogenesis, cytokine hypothesis, monoamine hypothesis, mesenchymal stem cells

## Abstract

Major Depressive Disorder (MDD), colloquially known as depression, is a debilitating condition affecting an estimated 3.8% of the population globally, of which 5.0% are adults and 5.7% are above the age of 60. MDD is differentiated from common mood changes and short-lived emotional responses due to subtle alterations in gray and white matter, including the frontal lobe, hippocampus, temporal lobe, thalamus, striatum, and amygdala. It can be detrimental to a person’s overall health if it occurs with moderate or severe intensity. It can render a person suffering terribly to perform inadequately in their personal, professional, and social lives. Depression, at its peak, can lead to suicidal thoughts and ideation. Antidepressants manage clinical depression and function by modulating the serotonin, norepinephrine, and dopamine neurotransmitter levels in the brain. Patients with MDD positively respond to antidepressants, but 10–30% do not recuperate or have a partial response accompanied by poor life quality, suicidal ideation, self-injurious behavior, and an increased relapse rate. Recent research shows that mesenchymal stem cells and iPSCs may be responsible for lowering depression by producing more neurons with increased cortical connections. This narrative review discusses the plausible functions of various stem cell types in treating and understanding depression pathophysiology.

## 1. Introduction

Major Depressive Disorder (MDD) is considered to be the most frequent psychiatric disorder [1] and, according to the World Health Organization (WHO), the leading cause of disability [2]. Low mood, diminished interest in daily activities, guilt, loss of pleasure, difficulty concentrating, low self-esteem, trouble sleeping, and altered appetite are some of the MDD symptoms. These issues can become chronic or recurrent, with severe consequences on a person’s ability to carry out daily activities. At its worst, depression can lead to suicidal thoughts [3]. Depression has been related to an increased chance of suffering from other severe illnesses, such as cardiovascular disease [4], stroke [3], Alzheimer’s disease [5], epilepsy [6], diabetes [7], and cancer [8]. Depressive symptoms are more commonly seen in older people, but this is due to factors linked with ageing, including physical disability [9], cognitive deficits, socioeconomic drawbacks, and other factors [10]. Treatment-resistant depression (TRD) can be caused by continuous exposure to environmental stressors during development [11].

Almost all antidepressants work the same way and effectively treat severe MDD across the lifespan [12,13]. However, antidepressant therapy has a number of adverse side effects, including sedation, headaches, decreased blood pressure, insomnia, weight gain, indigestion, feeling agitated, dry mouth, diarrhea, and sexual dysfunction [14]. This frequently leads to poor patient compliance, resulting in a recurrence of depressive symptoms and a higher risk of suicide [14].

Stem cells sustain the potential to help with tissue regeneration due to their capacity to self-renew and differentiate into numerous other cell types. In this narrative review, we explore the role of different stem cell types in managing, understanding, and treating depression.

## 2. Neurochemistry of Depression: The Monoamine Hypothesis

**Figure 1:**
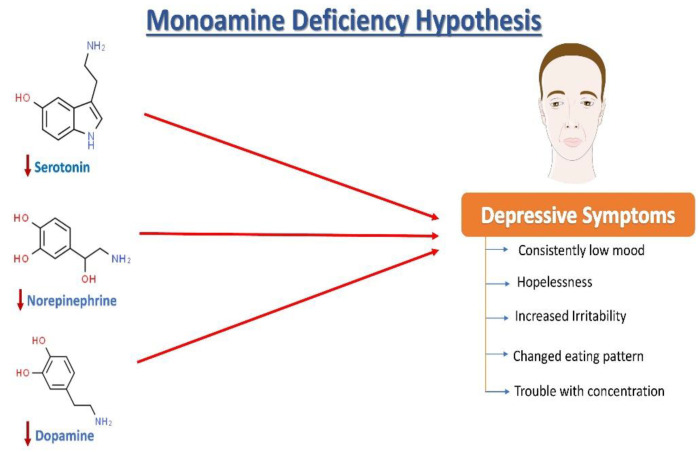
The monoamine hypothesis of depression. The reduced levels of serotonin, norepinephrine, and dopamine have been observed and are understood as one of the main factors responsible for the generation of depressive symptoms.

Norepinephrine (NE), serotonin (5-hydroxytryptamine, 5HT), and dopamine (DA) dysregulation are linked to the pathological changes seen in depression [15] (Figure 1). According to the monoamine hypothesis of depression, NE, 5HT, and DA work in synchrony to regulate emotions and mood [15]. In the depressed mood, the dysregulation of these three monoamines is observed, along with extracellular 5HT levels being lower than average. It has been reported that monoamines and metabolites are found to be lower in the urine, blood, and cerebrospinal fluid (CSF) of patients with depression as compared to age-matched controls [16].

The sympathetic nervous system’s primary neurotransmitter, NE, is produced in the locus coeruleus (LC) and is capable of sending projections all over the CNS. Research on depression began with a focus on the noradrenergic system, starting with introducing the “motor activation deficit” hypothesis [16]. Patients with depression and victims of suicidal behavior have been seen to show deficits in the noradrenergic system in the LC compared with healthy individuals [17]. Furthermore, some genetic changes in norepinephrine transporters (NETs) are highly plausible to be associated with psychiatric diseases [18,19]. It has been discovered that heterogeneously projecting neurons from the LC can separately lead to modulation of fear and learning, emphasizing that an imbalance in the activation of these neuronal groups may be linked with post-traumatic stress disorder (PTSD) and depression in rats [15]. Another monoamine neurotransmitter, DA, is well known to play an essential role in motivation, concentration, reward, psychomotor speed, and emotional response [15]. Notably, changes in DA functioning have also been linked to depression-related fatigue symptoms [20]. Depressed patients have lower DA metabolites in their CSF, which supports this hypothesis [21]. Pharmacologically, increased dopamine binding and availability of the dopamine transporter (DAT) in the striatum have been observed to improve depression symptoms in humans [22]. Furthermore, ropinirole, a dopaminergic targeting drug, has improved antidepressant treatment responsiveness in refractory patients [23].

Serotonin is synthesized in the dorsal raphe nucleus from tryptophan (via a 5-OH-tryptophan intermediate). The dorsal raphe nucleus sends projections to the whole CNS including, remarkably, the brain areas that are vulnerable to stress, such as the hippocampus. Serotonin is a widely distributed neurotransmitter that also acts as a neuromodulator [24,25,26]. Ever since its discovery, 5HT in the brain has been linked to circadian rhythms, sleep, cognitive abilities, appetite, motor activities, and many more biological functions [27]. Furthermore, 5HT involvement in response to stress [28] and psychiatric disease [29] has received a lot of attention. A decrease in serum 5HT [30] and plasma tryptophan [31] levels and alterations in metabolite levels of 5HIAA (5-hydroxyindoleacetic acid) in CSF have been observed in patients with a depressive illness [31]. Serotonin and its metabolites however have not been found to be a persistent biomarker of depression [26]. The function of 5HT receptors (particularly autoreceptors) and 5HT transporters is also being studied in patients with depression [26].

## 3. Growth Factors Involved in Depression

**Figure 2:**
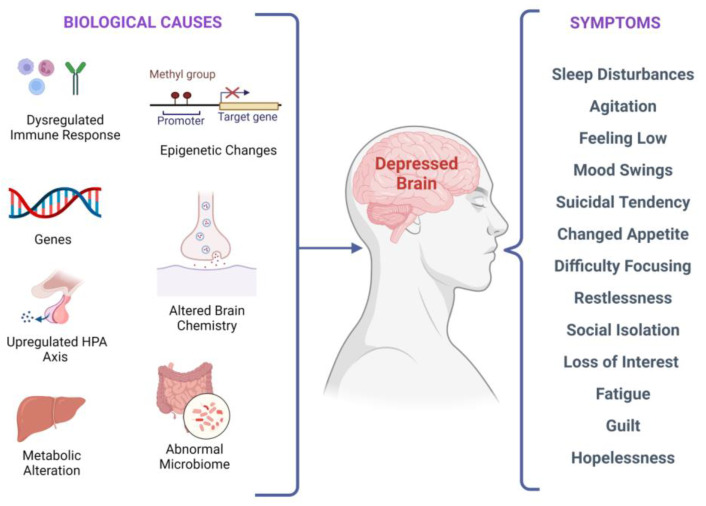
Biological factors and clinical manifestations associated with depression. There are multiple biological causes at molecular, genetic, epigenetic, cellular, and systems levels. These causes result in clinical depression and can have a plethora of symptoms that may vary in different individuals.

There are several biological factors associated with depressive symptoms, as shown in Figure 2. It has been investigated that stress-induced epigenetic changes can lead to depression [32,33]. Two meta-analyses of studies examining temporal lobe structures in MDD indicated that patients with recurrent depression have a smaller hippocampus [34,35]. Synaptic plasticity in neural circuits associated with depressive behaviors is regulated by the brain-derived neurotrophic factor (BDNF) [36,37,38]. Interestingly, stress-induced impairments in the brain structure and synaptic plasticity may be reversed by BDNF upregulation, leading to flexibility in cognition and an elevated capacity to acclimatize to environmental changes that might stimulate depressive episodes. According to current research, in depressed subjects, BDNF levels in the blood are lower, and they increase with antidepressant treatment [39]. Additionally, elevated BDNF plasma levels have been linked to better treatment outcomes regardless of the medication used [40].

Furthermore, anxiety, depression risk, neuroticism, and serotonergic neurotransmission have all been linked to altered serum BDNF levels and BDNF gene polymorphism [2]. Lithium augmentation refers to the addition of lithium to an antidepressant in the acute treatment phase of depression [41,42]. Antidepressant augmentation with lithium is a well-studied augmentation therapy for patients with depression who have not been responding well to antidepressant therapy [41]. Moreover, lithium augmentation has the ability to increase BDNF concentrations in the serum [40].

The reduced levels of nerve growth factor (NGF) are involved in the pathophysiology of depression. Fluoxetine and lithium are known to treat depression by upregulating NGF protein levels in the hippocampus [43]. Neurotrophin-3 (NT-3) is responsible for neuron proliferation, differentiation, and survival. NT-3 also promotes the growth of axons and dendrites. Clinical data from post-mortem research showed that people with depressive disorders have lower NT-3 levels in their parietal brain [44,45]. The decreased glial cell line-derived neurotrophic factor (GDNF) in the serum is correlated with the development of depressive symptoms. GDNF has a crucial role in the survival and maintenance of monoaminergic neurons. It has been reported that reduced GDNF levels abnormally regulate serotonergic neurons and reduce post and pre-synaptic serotonin receptors in patients with depression [46]. Fibroblast growth factor-2 (FGF-2) is another growth factor associated with depression [47]. Recent research has suggested that BDNF may signal via the Akt and GSK3 pathways [48].

Further, it has been seen that erythropoietin impacts neuroplasticity and could be used to treat depression in the future. A study by Miskowiak et al. recruited 40 patients with a bipolar mood disorder and 40 patients with TRD. The patients received either a weekly intravenous infusion of erythropoietin or saline. It was observed that a single dose of erythropoietin (Eprex; 40,000IU) could improve cognitive functioning and reduce the neurocognitive processing involved in negative emotional information in normal versus people with depression in a way that is similar to antidepressants’ effects [49]. In the limited research conducted on unipolar depression, inositol, a component of the intracellular phosphatidyl-inositol second-messenger system, has also been shown to be effective [50]. Finally, peripheral VGF (nonacronymic) showed a reduced expression in MDD patients, and recombinant VGF administration causes antidepressant effects in rats [50].

## 4. Depression as a Neurodegenerative Disorder

Neurodegenerative disorders are conditions in which the central nervous system cells stop functioning or die [51,52,53]. The majority of neurodegenerative diseases are incurable and often worsen with time [54,55]. Patients suffering from depression have been shown to have a reduced cortical thickness in the right superior temporal, left inferior temporal lobe, and right pars orbitalis in comparison to healthy controls [56]. Temporal and prefrontal cortex structural alteration has also been reported in people with both depression and anxiety. The atrophy seen in the prefrontal and temporal cortices may be a typical pattern of cortical and subcortical changes [57]. The hippocampus, one of the most explored limbic structures, is thought to be the integrator of emotional response and cognition. It is involved in the development of new memories and guides our behavioral response by comparing new stimulatory inputs with the memories which have been previously stored [58]. On the other hand, caudate nucleus volume alteration may indicate anxious depression, which can be used to differentiate anxious from non-anxious depression [56]. Many studies have used neuroimaging methods, particularly magnetic resonance imaging (MRI), to identify structural changes in the brain linked with MDD in recent years.

High-resolution structural imaging, which shows grey matter thickness and brain morphology; diffusion tensor imaging (DTI), which can record the white matter and its microstructures; and functional MRI (fMRI), which shows the neuronal activity in specific brain areas, are all MRI scan sequences that researchers commonly use. MRI studies have previously revealed that patients with MDD have significant impairments in numerous brain regions. The frontal and parietal lobes, thalamus, caudate, pallidum, putamen, and temporal lobes have shown regional grey matter changes (e.g., the hippocampus and amygdala) in these patients [57]. The frontal lobe is considered the most typical region showing anatomic abnormalities in depression [59]. Studies have shown that the thickness of the prefrontal region is reduced, and these changes are linked with poor clinical outcomes in depression [56]. Moreover, depression shows some of the same pathological features as seen in neurodegenerative disorders such as Alzheimer’s disease [60]. In both depression and neurodegenerative disorders, neuroinflammation is observed. The monoamine oxidase pathways are implicated in depression, Parkinson’s disease, and Alzheimer’s disease [60]. Therefore, several monoamine oxidase inhibitors are used to treat neurodegenerative disorders and depression. Furthermore, hypothalamus–pituitary axis dysfunction is seen in both neurodegenerative disorders and depression [60].

## 5. Stem Cells: An Insight

Undifferentiated, pluripotent cell types known as stem cells are found in the embryonic, fetal, and adult stages of almost all organisms. They have the ability to develop into different types of differentiated cells as per the spatial and temporal distribution. These cells can be classified based on differentiation and origin. The stem cells that are specific for tissues are found in organs, which have been differentiated in the postnatal and adult lifespans and have an essential role in organ repair after injury. Self-renewal, clonality, and potency are crucial features of stem cells [60].

**Figure 3:**
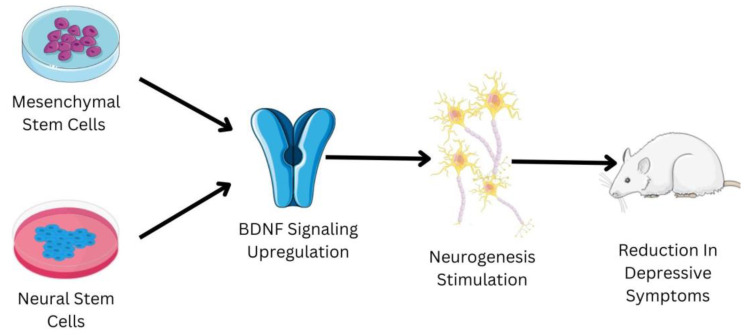
Shows the role of mesenchymal stem cells (MSCs) and neural stems cells (NSCs) in the management of depression. The reduction in the depressive symptoms in the mice animal model has been observed due to administration of MSCs and NSCs. The MSCs and NSCs have the ability to upregulate the BDNF signaling pathway, which is usually downregulated in patients suffering from depression. The upregulation of the BDNF signaling pathway helps in the stimulation of neurogenesis. Neurogenesis is one of the major biological factors responsible for the development of depression; the increase in neurogenesis helps in relieving the symptoms associated with depression.

Embryonic stem cells are derived from the zygote and blastocyst to create the three germ layers: endoderm, mesoderm, and ectoderm. The germ layers mature into a specific organ. Tissue stem cells can be present in bone marrow, bone, liver, brain, etc., since certain progenitor cells that contributed to organogenesis do not differentiate terminally [61]. Since they inevitably lead to differentiated cells and specialized cells of the tissue or organ, tissue stem cells are also known as progenitor cells. These cells may be quiescent within the tissue, but they will multiply in the case of an injury [62]. In the 1950s, the first trials of bone marrow transplants in animal models led to the start of contemporary medicine’s studies with stem cells and organ repair [63]. These pioneering experiments paved the way for human bone marrow transplantation, which is a common treatment for a variety of blood diseases, and established a unique therapeutic method for tissue regeneration with the use of stem cells. Regenerative medicine is a significant focus of research, not just in terms of seeking solutions but also in terms of understanding basic biology and disease causation [64]. Regenerative medicine links and combines various fields, including engineering, technology, biology, and medicine, intending to restore tissue homeostasis by repairing or replacing damaged cells, tissues, or organs [65]. Despite the fact that research into stem cells has raised a number of ethical concerns, advances in stem cell isolation and development have allowed scientists to identify culture-specific cell types for tissue repair in several disorders [66]. Stem cells have the ability to treat depression; a schematic shown in Figure 3 briefly elucidates the mechanism of some of the types of stem cells to treat depression.

## 6. Intranasal Route for Stem Cells Administration

Stem cells therapy has crucial clinical properties in the treatment of disorders associated with CNS. One of the major obstacles in the delivery of stem cells or any type of drug into the CNS is the blood brain barrier (BBB) [67,68]. The use of stereotactic or intrathecal injections is usually necessary for stem cell therapy in the brain. The invasive procedure restricts the use of stem cell transplantation in the treatment of neurological conditions [67]. Numerous studies have shown that an intranasal application (INA) can circumvent the BBB and transfer small molecules and macromolecules to the CNS. Furthermore, INA is less invasive and more reliable than stereotactic brain injections and intrathecal injections. INA has no disease specificity, but it also demonstrates the benefits of its broad use across a range of neurological disorders. INA is preferable over the intravenous administration in a number of ways: (1) it delivers therapeutic agents to the CNS to a large extent; (2) it manages to avoid first-pass metabolism; (3) it is noninvasive and simple to use, allowing for repeated administration if required; and (4) its side effects are reduced since no other healthy organs are exposed to the therapeutic agent [67]. The intranasal injection of neural stem cells (NSCs), mesenchymal stem cells (MSCs), and induced pluripotent stem cells (iPSCs) have been used in the treatment of various neurological disorders [68]. The INA of MSCs have been widely used in the research and treatment of glioma, neurodegeneration, and brain injury. The INA iPSCs have also been used for studies related to chronic inflammation. In INA, substances accumulate in the nasal cavity’s epithelial cell layer and go straight to the brain parenchyma along the olfactory or trigeminal nerves or via the cerebrospinal fluid system. The intracellular and paracellular transport channels can transfer the substances to the CNS once they have arrived at the targeted site [67].

## 7. Utilizing the Induced Pluripotent Stem Cells (iPSCs) to Understand Depression Pathophysiology

Adult somatic cells have been transformed genetically to an embryonic stem (ES) cell-like state through enforced genetic expression and proteins crucial for preserving the defining traits of ES cells, resulting in iPSCs [68]. Human iPSCs (hiPSCs) allow for the recreation of cellular phenotypes, typically in depression and the search for new antidepressant treatments. Moreover, hiPSCs are used for the verification of existing antidepressant efficacy, and to provide new information about depression etiopathogenesis [69]. Hedgehog signaling irregularities have been reported in the hiPSCs of people with bipolar depression, indicating that this aberration can be potentially linked to depression [69]. Despite the monoaminergic hypothesis’ oversimplification, depression’s recent pharmacological treatment relies on medications that primarily target the monoamine neurotransmitter systems. This has a delayed efficacy with a lag duration of several weeks to months before exhibiting clinical improvement [70]. Psychiatry, on the other hand, is going through a fascinating period. The hiPSC technology, when combined with current breakthroughs in genome editing tools, offers innovative and distinct options in disease modeling and medication discovery. For many psychiatric diseases, this technique has enabled the creation of new disease-relevant patient-specific in vitro models [71]. These models promise to improve our understanding of the pathophysiology of patients with MDD while also addressing many of the known practical constraints of animal and post-mortem models [72]. Interestingly, Marcatili et al. used the hiPSC technology-based TRD model to study ketamine’s mechanism of action as an antidepressant, which might contribute to customized and less hazardous new treatments [70]. Furthermore, two distinct research groups have advanced iPSC research, allowing this technology to be applied to the field of depression study by using the iPSC technique to generate serotonergic neurons. In vitro studies of neurons are therefore now possible. Previously, only animal models were used to study neurotransmission [73]. In two recent papers [74,75], serotonergic neuron generation by harnessing iPSCs has been reported. Antidepressants, notably SSRIs such as fluoxetine, sertraline, citalopram, paroxetine, and escitalopram, target serotonergic neurons that are dysregulated in depression. Serotonergic neurons may therefore be studied now using new techniques in patients with depression [73].

## 8. Mesenchymal Stem Cells and Depression: A Therapeutic Mechanism

MSCs are a diverse kind of stromal stem cell that can be separated from a variety of adult tissues [75,76], such as the umbilical cord, endometrial polyps, bone marrow, adipose tissue, and others [77]. They can segregate into mesodermal lineage cells such as osteocytes, adipocytes, chondrocytes, and some other embryonic lineages [75]. The presence of MSCs has also been recently observed in other sources, such as menstrual blood. These MSCs prove to be a noteworthy option for future clinical and experimental applications, and there are likely more sources of MSCs yet to be discovered. One of the most difficult tasks is to elucidate the mechanisms of MSC differentiation, mobilization, and homing. MSCs’ multipotent properties make them a considerate option for clinical application development [77]. Several studies have also observed the anti-inflammatory activity of MSCs [78]. Notably, MSCs have the ability to downregulate the expression of the proinflammatory cytokines IL-1β, IL-6, and TNF-α [79], which is one of the major reasons that contribute to the development of depression, as discussed in the cytokine hypothesis. Zhang et al. studied the antidepressant-like effect of human umbilical cord mesenchymal stem cells (HUC-MSCs) on microglial polarization and depression-like symptoms associated with myocardial infarction (MI). They concluded that injecting HUC-MSCs in the seven-week-old male mice model significantly improves cardiac function and depression-like behavior caused by MI. This was achieved through Jmjd3 level downregulation and M1/M2 microglia polarization regulation. HUC-MSCs can bring more important benefits to patients with depression and MI compared with typical antidepressants [79]. Most antidepressants work on modulating monoamine transmission, yet numerous patients experience low residual symptoms and remission rates. Recent research suggests that glutamatergic abnormalities and glial pathology, as well as the monoaminergic system, play a major role in the etiology and manifestation of symptoms associated with depression [80]. Altered levels of glutamate in plasma [81], serum [82,83], CSF [84], and brain tissue were observed in suicide victims and people with mood disorders [85,86,87]. Animal models of induced depression, such as the genetic mice model for depression, Flinders Sensitive Line (FSL), show glutamatergic impairments. FSL mice also show increased resting glutamate levels and glutamate transients in the prefrontal cortex [88]. Importantly, the drop in glial numbers is a common neuropathological finding in MDD [77,88,89], which has the potential to reduce neuronal plasticity. FSLs exhibit defective astrocytic regulation of glutamate transmission in the hippocampus [90], including downregulation of the glial excitatory amino acid transporter (EAAT) 1, a key member of the glutamate/neutral amino acid transporter protein family [90]. Several glutamatergic drugs have been proposed as potential antidepressants, while their sedative and psychotomimetic side effects may limit their usage [91]. The study by Shwartz et al. found that when differentiating human MSCs expressing high levels of EAAT1 and EAAT2 were administered via intracerebroventricular injection in FSL mice, it showed a long-term depressive-like behavior attenuating impact on these animals, affecting motivation, novelty exploration, and hedonia. In recent research, nanoparticles (NPs) are being used as a theranostic tool for the treatment and diagnosis of several neurological and mood disorders [92]. Using non-invasive, real-time imaging of the gold nanoparticles (GNPs) that labelled the cells, followed by a quantitative analysis of gold amounts in the brain regions, it was discovered that the majority of EAAT-positive MSCs moved to the dentate gyrus of the hippocampus, and were identified in this region up to one-month after transplantation, [93]. The behavioral effect of MSC-EAATs on FSLs appears to follow a pattern similar to that of other pharmacological and non-pharmacological anti-depressant treatments, with the strongest effect occurring within 2–3 weeks of treatment, when a balance in the expression of depression-related receptors (e.g., dopamine and 5-HT) is achieved [93]. Thus, treatment with MSC-EAAT may improve depressive-like behaviors by restoring normal hippocampus glutamatergic transmission and BDNF levels. This idea was confirmed by a subsequent study conducted by Nibuya et al. that found defective astrocytic glutamate regulation in the hippocampus of FSLs, including the downregulation of glia EAAT1 expression [93].

Further, excessive proinflammatory cytokines release such as MCP-1, IL-1, IL-6, and TNF-alpha leads to behaviors comparable to depression [94]. Recently, it was discovered that after depression was induced, MCP-1, IL-1, IL-6, and TNF expression increased significantly. Expression of MCP-1, IL-1, IL-6, and TNF- was decreased by adipose-derived mesenchymal stem cells (ADSC) therapy. These ADSCs were extracted and grown from adipose tissues (mouse abdominal fat) for the study [95]. These stem cells are multipotent and capable of differentiating into a variety of cell types, including those of adipogenic, myogenic, chondrogenic, and osteogenic lineage [96]. The findings also demonstrated that ADSC treatment decreased mice’s depressive-like behaviors in the sucrose preference test (SPT), tail suspension test (TST), and forced swimming test (FST), which is consistent with earlier depression and inflammation studies [97]. It was also shown that ADSC treatment enhanced the levels of BDNF and TrkB expression, which had previously been observed to be reduced following depression. The BDNF-TrkB signaling pathway has been previously demonstrated to modulate brain inflammation and protect against hippocampus injury, suggesting that ADSC-mediated protective effects could be linked to reduced symptoms of depression [98,99]. ADSCs have the ability to engraft in the brain tissue and develop into neurons and glial cells following transplantation, and as a result, they are commonly used in peripheral nerve regeneration [100]. ADSC transplantation therapy has been demonstrated to have anti-inflammatory benefits in recent investigations [101]. Further research revealed that depression increased TLR4/NF-B activation while simultaneously suppressing the Nrf2/HO-1 signaling pathway. ADSC therapy, on the other hand, enhanced Nrf2/HO-1 signaling while decreasing TLR4/NF-B activation. TLR4 signaling activates the JNK signaling cascade, causing neuroinflammation and neurodegeneration, and/or interacts with the Bcl-2 family of proteins, causing the mitochondrial apoptotic pathway to be activated [102]. It has been observed that depression is associated with a decrease in neurogenesis. MSCs have the ability to stimulate neurogenesis by neurotrophic factor expression and differentiate into neural lineages [103]. In another study by Tfilin et al., MSCs were derived from the adult bone marrow and were injected into FSL rats (an animal model for depression) through a cerebroventricular injection. The MSC-transplanted rats showed improvement in behavioral performance when measured with the dominant–submissive relationship (DSR) paradigm and forced swim test [103]. After the transplantation, the MSCs will start migrating the dentate gyrus, CA3, and CA1 regions of the hippocampus, hypothalamus, thalamus, and contralateral hippocampus, and neurogenesis will be stimulated. Neurogenesis helps in modulating the treated depressive disorders [103]. Furthermore, a study performed by Kin et al. explained that the administration of encapsulated MSCs into the lateral ventricle of Wistar Kyoto (WKY) rats, which are promising animal models of TRD, exerts antidepressant effects. The implantation of encapsulated MSCs is associated with the upregulation of the intrinsic expression of CNTF and VEGF and their receptors [104].

## 9. Neural Stem Cells and Depression

NSCs have garnered interest in recent years with the extensive published literature elucidating that the adult brain maintains multipotent NSCs in contrast to the old dogma of the brain being a generally invariable and quiescent organ that lacks the flexibility to regenerate. With their most generally accepted distinguishing traits, NSCs are also ascribed to the so-called tissue stem cells [105], featuring the power to stay undifferentiated without an outlined phenotype under specific conditions, the power of dividing and proliferating (self-renewal), and also the ability to be differentiated into a progeny like neurons, oligodendroglia, and astroglia upon neurogenesis initiation. They are the unique types of competent cells found within the adult mammalian brain’s “neurogenic” regions, such as the hippocampus [106], subventricular zone [107], and neural structures [108], and might create neurons both spontaneously and in response to local signals induction. Neurogenesis (NG) is assumed to need an explicit set of signaling cues to be delivered to cells that are neurogenic in a very spatially and temporally coordinated manner by their surroundings so as to activate stem cells or progenitors to develop new neurons and, in addition to the well-known modulators [109], injury is considered to be sufficient to activate neurogenesis. Neurogenesis is also stimulated by the expression of BDNF [110]. NSCs are often extracted from adult brain tissues, including post-mortem brain tissue [111], and become significant candidates for increasing or restoring the quality and function of brain tissue affected with CNS-related illnesses. The NSCs are clonally expanded in vitro, genetically manipulated, or stimulated to transform CNS cell lineages [111]. Understanding how adult neurogenesis is regulated has required significant work. Because of this, we now understand that numerous intrinsic and extrinsic pathways might influence this process [111].

Growth factors, transmitters, enzymes, tissue hormones, neuromodulators, and antibodies are predicted to be secreted into the local tissue environment by activated cells, eliciting desirable tissue responses. In damaged neuronal and glial networks, the newly empowered cells and their progeny can operate as functional enhancers and scaffold “healing agents.” These properties have led to substantial advancements in the invention of therapies for trauma and perfusion issues such as stroke [112], ischemia, or neurodegeneration-related conditions [112,113,114]. Not unexpectedly, the prospects of NSCs in mental health care are being hotly debated. Numerous psychiatric illnesses are likely to possess genetic variants and specific cellular and anatomical correlations that are mostly unknown [112].

In depression, a reduction of neurogenesis is often seen in the hippocampus [115]. This further implies that neurogenesis deficiencies might cause the symptoms associated with depression, while enhanced neurogenesis can mediate antidepressant action and ease symptoms. However, various conflicting reports regarding the role of neurogenesis in alleviating depression must be first reconciled before this bidirectional concept’s complete legitimacy is established [116]. The activation of adult hippocampal neurogenesis leads to the transformation of neural somatic cell progeny to mature CNS neurons. These CNS neurons then acquire functional and morphological qualities to integrate into existing neural networks or replace various other brain cells that have died [117,118].

## 10. Conclusions

Recent pharmacological treatments of depression rely on medications that primarily target monoamine neurotransmitter systems. This has a delayed efficacy with lag durations of several weeks to months before exhibiting clinical improvement. Psychiatry, on the other hand, is going through a fascinating period. Importantly when combined with current breakthroughs in genome editing tools, hiPSC technology offers innovative and distinct options in disease modeling and medication discovery for depression. Reduced hippocampal neurogenesis has been observed in depression. This implies that NG deficiencies might cause depressed symptoms of depression and that enhanced NG can mediate antidepressant action and ease symptoms. Moreover, as reported in experimental models, the differentiated MSCs have a promising treatment capability in reversing depressive-like behavior.

## Data Availability

Not applicable.

## Abbreviations

5HT	5-hydroxytryptamine
ADSCs	Adipose-derived mesenchymal stem cells
BBB	Blood brain barrier
BDNF	Brain-derived neurotrophic factor
CNS	Central nervous system
CRP	C-reactive protein
CSF	Cerebrospinal fluid
DA	Dopamine
DAT	Dopamine transporter
DTI	Diffusion tensor imaging
BBB	Blood-brain barrier
EAAT	Excitatory amino acid transporter
FGF-2	Fibroblast growth factor-2
fMRI	functional MRI
FSL	Flinders sensitive line
FST	Forced swimming test
GDNF	Glial cell line-derived neurotrophic factor
GNPs	Gold nanoparticles
hiPSCs	Human iPSCs
HUC-MSCs	Human umbilical cord mesenchymal stem cells
iPSCs	Induced pluripotent stem cells
LC	Locus coeruleus
MDD	Major depressive disorder
MI	Myocardial Infarction
MRI	Magnetic resonance imaging
MSCs	Mesenchymal stem cells
NE	Norepinephrine
NETs	Norepinephrine transporters
NG	Neurogenesis
NGF	Nerve growth factor
NSCs	Neural stem cells
NT	3-Neurotrophin-3
PGE2	Plasma prostaglandin E2
PTSD	Post-traumatic stress disorder
SNRIs	Serotonin-norepinephrine reuptake inhibitors
SPT	Sucrose preference test
SSRIs	Selective serotonin reuptake inhibitors
TLR	Toll-like receptor
TRD	Treatment-resistant depression
TST	Tail suspension test
VGF	nonacronymic

## References

[1] Maj M. (2011). When Does Depression Become a Mental Disorder?. Br. J. Psychiatry.

[2] Lang U.E., Borgwardt S. (2013). Molecular Mechanisms of Depression: Perspectives on New Treatment Strategies. Cell Physiol. Biochem..

[3] Ramasubbu R., Patten S.B. (2003). Effect of Depression on Stroke Morbidity and Mortality. Can. J. Psychiatry.

[4] Van der Kooy K., van Hout H., Marwijk H., Marten H., Stehouwer C., Beekman A. (2007). Depression and the Risk for Cardiovascular Diseases: Systematic Review and Meta Analysis. Int. J. Geriat. Psychiatry.

[5] Green R.C., Cupples L.A., Kurz A., Auerbach S., Go R., Sadovnick D., Duara R., Kukull W.A., Chui H., Edeki T. (2003). Depression as a Risk Factor for Alzheimer Disease: The MIRAGE Study. Arch. Neurol..

[6] Hesdorffer D.C., Hauser W.A., Annegers J.F., Cascino G. (2000). Major Depression Is a Risk Factor for Seizures in Older Adults. Ann. Neurol..

[7] Nouwen A., Lloyd C.E., Pouwer F. (2009). Depression and Type 2 Diabetes Over the Lifespan: A Meta-Analysis. Diabetes Care.

[8] Penninx B.W.J.H., Guralnik J.M., Havlik R.J., Pahor M., Ferrucci L., Cerhan J.R., Wallace R.B. (1998). Chronically Depressed Mood and Cancer Risk in Older Persons. JNCI J. Natl. Cancer Inst..

[9] Kaur J., Ghosh S., Singh P., Dwivedi A.K., Sahani A.K., Sinha J.K. (2022). Cervical Spinal Lesion, Completeness of Injury, Stress, and Depression Reduce the Efficiency of Mental Imagery in People With Spinal Cord Injury. Am. J. Phys. Med. Rehabil..

[10] Maniam J., Antoniadis C.P., Youngson N.A., Sinha J.K., Morris M.J. (2015). Sugar Consumption Produces Effects Similar to Early Life Stress Exposure on Hippocampal Markers of Neurogenesis and Stress Response. Front. Mol. Neurosci..

[11] Rush A.J., Trivedi M.H., Wisniewski S.R., Nierenberg A.A., Stewart J.W., Warden D., Niederehe G., Thase M.E., Lavori P.W., Lebowitz B.D. (2006). Acute and Longer-Term Outcomes in Depressed Outpatients Requiring One or Several Treatment Steps: A STAR*D Report. Am. J. Psychiatry.

[12] Akil H., Gordon J., Hen R., Javitch J., Mayberg H., McEwen B., Meaney M.J., Nestler E.J. (2018). Treatment Resistant Depression: A Multi-Scale, Systems Biology Approach. Neurosci. Biobehav. Rev..

[13] Salzman C., Wong E., Wright B.C. (2002). Drug and ECT Treatment of Depression in the Elderly, 1996–2001: A Literature Review. Biol. Psychiatry.

[14] Keller M.B., Hirschfeld R.M.A., Demyttenaere K., Baldwin D.S. (2002). Optimizing Outcomes in Depression: Focus on Antidepressant Compliance. Int. Clin. Psychopharmacol..

[15] Nestler E.J., Carlezon W.A. (2006). The Mesolimbic Dopamine Reward Circuit in Depression. Biol. Psychiatry.

[16] Roy A. (1989). Cerebrospinal Fluid Monoamine Metabolites and Suicidal Behavior in Depressed Patients: A 5-Year Follow-up Study. Arch. Gen. Psychiatry.

[17] Klimek V., Stockmeier C., Overholser J., Meltzer H.Y., Kalka S., Dilley G., Ordway G.A. (1997). Reduced Levels of Norepinephrine Transporters in the Locus Coeruleus in Major Depression. J. Neurosci..

[18] Marshe V.S., Maciukiewicz M., Rej S., Tiwari A.K., Sibille E., Blumberger D.M., Karp J.F., Lenze E.J., Reynolds C.F., Kennedy J.L. (2017). Norepinephrine Transporter Gene Variants and Remission From Depression With Venlafaxine Treatment in Older Adults. Am. J. Psychiatry.

[19] Dunn A.J. (2006). Effects of Cytokines and Infections on Brain Neurochemistry. Clin. Neurosci. Res..

[20] Anacker C., Cattaneo A., Musaelyan K., Zunszain P.A., Horowitz M., Molteni R., Luoni A., Calabrese F., Tansey K., Gennarelli M. (2013). Role for the Kinase SGK1 in Stress, Depression, and Glucocorticoid Effects on Hippocampal Neurogenesis. Proc. Natl. Acad. Sci. USA.

[21] Jokinen J., Nordström A.-L., Nordström P. (2007). The Relationship Between CSF HVA/5-HIAA Ratio and Suicide Intent in Suicide Attempters. Arch. Suicide Res..

[22] Pizzagalli D.A., Berretta S., Wooten D., Goer F., Pilobello K.T., Kumar P., Murray L., Beltzer M., Boyer-Boiteau A., Alpert N. (2019). Assessment of Striatal Dopamine Transporter Binding in Individuals With Major Depressive Disorder: In Vivo Positron Emission Tomography and Postmortem Evidence. JAMA Psychiatry.

[23] Cassano P., Lattanzi L., Fava M., Navari S., Battistini G., Abelli M., Cassano G.B. (2005). Ropinirole in Treatment-Resistant Depression: A 16-Week Pilot Study. Can. J. Psychiatry.

[24] Descarries L., Watkins K.C., Garcia S., Beaudet A. (1982). The Serotonin Neurons in Nucleus Raphe Dorsalis of Adult Rat: A Light and Electron Microscope Radioautographic Study. J. Comp. Neurol..

[25] Bunin M.A., Wightman R.M. (1998). Quantitative Evaluation of 5-Hydroxytryptamine (Serotonin) Neuronal Release and Uptake: An Investigation of Extrasynaptic Transmission. J. Neurosci..

[26] Steinbusch H.W.M. (1981). Distribution of Serotonin-Immunoreactivity in the Central Nervous System of the Rat—Cell Bodies and Terminals. Neuroscience.

[27] Mann J. (1999). Role of the Serotonergic System in the Pathogenesis of Major Depression and Suicidal Behavior. Neuropsychopharmacology.

[28] Chaouloff F. (1999). Serotonin and Stress. Neuropsychopharmacology.

[29] Andrews P.W., Bharwani A., Lee K.R., Fox M., Thomson J.A. (2015). Is Serotonin an Upper or a Downer? The Evolution of the Serotonergic System and Its Role in Depression and the Antidepressant Response. Neurosci. Biobehav. Rev..

[30] Bot M., Chan M.K., Jansen R., Lamers F., Vogelzangs N., Steiner J., Leweke F.M., Rothermundt M., Cooper J., Bahn S. (2015). Serum Proteomic Profiling of Major Depressive Disorder. Transl. Psychiatry.

[31] Quintana J. (1992). Platelet Serotonin and Plasma Tryptophan Decreases in Endogenous Depression. Clinical, Therapeutic, and Biological Correlations. J. Affect. Disord..

[32] Park C., Rosenblat J.D., Brietzke E., Pan Z., Lee Y., Cao B., Zuckerman H., Kalantarova A., McIntyre R.S. (2019). Stress, Epigenetics and Depression: A Systematic Review. Neurosci. Biobehav. Rev..

[33] Ghosh S., Sinha J.K., Raghunath M. (2019). “Obesageing”: Linking Obesity & Ageing. Indian J. Med. Res..

[34] Campbell S., MacQueen G. (2006). An Update on Regional Brain Volume Differences Associated with Mood Disorders. Curr. Opin. Psychiatry.

[35] Videbech P. (2004). Hippocampal Volume and Depression: A Meta-Analysis of MRI Studies. Am. J. Psychiatry.

[36] Mishra P., Mittal A.K., Kalonia H., Madan S., Ghosh S., Sinha J.K., Rajput S.K. (2021). SIRT1 Promotes Neuronal Fortification in Neurodegenerative Diseases through Attenuation of Pathological Hallmarks and Enhancement of Cellular Lifespan. Curr. Neuropharmacol..

[37] Moroi K., Sato T. (1975). Comparison between Procaine and Isocarboxazid Metabolism in Vitro by a Liver Microsomal Amidase-Esterase. Biochem. Pharmacol..

[38] Pittenger C., Duman R.S. (2008). Stress, Depression, and Neuroplasticity: A Convergence of Mechanisms. Neuropsychopharmacology.

[39] Aydemir O., Deveci A., Taneli F. (2005). The Effect of Chronic Antidepressant Treatment on Serum Brain-Derived Neurotrophic Factor Levels in Depressed Patients: A Preliminary Study. Prog. Neuro-Psychopharmacol. Biol. Psychiatry.

[40] Ricken R., Adli M., Lange C., Krusche E., Stamm T.J., Gaus S., Koehler S., Nase S., Bschor T., Richter C. (2013). Brain-Derived Neurotrophic Factor Serum Concentrations in Acute Depressive Patients Increase During Lithium Augmentation of Antidepressants. J. Clin. Psychopharmacol..

[41] Bauer M., Adli M., Bschor T., Pilhatsch M., Pfennig A., Sasse J., Schmid R., Lewitzka U. (2010). Lithium’s Emerging Role in the Treatment of Refractory Major Depressive Episodes: Augmentation of Antidepressants. Neuropsychobiology.

[42] Coradduzza D., Garroni G., Congiargiu A., Balzano F., Cruciani S., Sedda S., Nivoli A., Maioli M. (2022). MicroRNAs, Stem Cells in Bipolar Disorder, and Lithium Therapeutic Approach. Int. J. Mol. Sci..

[43] Mondal A.C., Fatima M. (2019). Direct and Indirect Evidences of BDNF and NGF as Key Modulators in Depression: Role of Antidepressants Treatment. Int. J. Neurosci..

[44] de Miranda A.S., de Barros J.L.V.M., Teixeira A.L. (2020). Is Neurotrophin-3 (NT-3): A Potential Therapeutic Target for Depression and Anxiety?. Expert Opin. Ther. Targets.

[45] Diniz B.S., Teixeira A.L., Miranda A.S., Talib L.L., Gattaz W.F., Forlenza O.V. (2012). Circulating Glial-Derived Neurotrophic Factor Is Reduced in Late-Life Depression. J. Psychiatr. Res..

[46] Evans S.J., Choudary P.V., Neal C.R., Li J.Z., Vawter M.P., Tomita H., Lopez J.F., Thompson R.C., Meng F., Stead J.D. (2004). Dysregulation of the Fibroblast Growth Factor System in Major Depression. Proc. Natl. Acad. Sci. USA.

[47] Beaulieu J.-M. (2012). A Role for Akt and Glycogen Synthase Kinase-3 as Integrators of Dopamine and Serotonin Neurotransmission in Mental Health. J. Psychiatry Neurosci..

[48] Miskowiak K.W., Vinberg M., Harmer C.J., Ehrenreich H., Knudsen G.M., Macoveanu J., Hansen A.R., Paulson O.B., Siebner H.R., Kessing L.V. (2010). Effects of Erythropoietin on Depressive Symptoms and Neurocognitive Deficits in Depression and Bipolar Disorder. Trials.

[49] Eden Evins A., Demopulos C., Yovel I., Culhane M., Ogutha J., Grandin L.D., Nierenberg A.A., Sachs G.S. (2006). Inositol Augmentation of Lithium or Valproate for Bipolar Depression. Bipolar Disord..

[50] Cattaneo A., Sesta A., Calabrese F., Nielsen G., Riva M.A., Gennarelli M. (2010). The Expression of VGF Is Reduced in Leukocytes of Depressed Patients and It Is Restored by Effective Antidepressant Treatment. Neuropsychopharmacology.

[51] Ahmad F., Sachdeva P., Sarkar J., Izhaar R. (2022). Circadian Dysfunction and Alzheimer’s Disease—An Updated Review. Aging Med..

[52] Ahmad F., Sachdeva P. (2022). Critical Appraisal on Mitochondrial Dysfunction in Alzheimer’s Disease. Aging Med..

[53] Mukerjee N., Al-Khafaji K., Maitra S., Suhail Wadi J., Sachdeva P., Ghosh A., Buchade R.S., Chaudhari S.Y., Jadhav S.B., Das P. (2022). Recognizing Novel Drugs against Keap1 in Alzheimer’s Disease Using Machine Learning Grounded Computational Studies. Front. Mol. Neurosci..

[54] Sachdeva B., Sachdeva P. (2023). MXenes for Neurodegenerative Disorders. Mater. Today: Proc..

[55] Madar I.H., Sultan G., Tayubi I.A., Hasan A.N., Pahi B., Rai A., Sivanandan P.K., Loganathan T., Begum M., Rai S. (2021). Identification of Marker Genes in Alzheimer’s Disease Using a Machine-Learning Model. Bioinformation.

[56] Zhao K., Liu H., Yan R., Hua L., Chen Y., Shi J., Lu Q., Yao Z. (2017). Cortical Thickness and Subcortical Structure Volume Abnormalities in Patients with Major Depression with and without Anxious Symptoms. Brain Behav..

[57] Nagy C., Maitra M., Tanti A., Suderman M., Théroux J.-F., Davoli M.A., Perlman K., Yerko V., Wang Y.C., Tripathy S.J. (2020). Single-Nucleus Transcriptomics of the Prefrontal Cortex in Major Depressive Disorder Implicates Oligodendrocyte Precursor Cells and Excitatory Neurons. Nat. Neurosci..

[58] Crane N.A., Jenkins L.M., Dion C., Meyers K.K., Weldon A.L., Gabriel L.B., Walker S.J., Hsu D.T., Noll D.C., Klumpp H. (2016). Comorbid Anxiety Increases Cognitive Control Activation in Major Depressive Disorder: Crane et Al. Depress. Anxiety.

[59] Zhang F.-F., Peng W., Sweeney J.A., Jia Z.-Y., Gong Q.-Y. (2018). Brain Structure Alterations in Depression: Psychoradiological Evidence. CNS Neurosci. Ther..

[60] Hussain M., Kumar P., Khan S., Gordon D.K., Khan S. (2020). Similarities Between Depression and Neurodegenerative Diseases: Pathophysiology, Challenges in Diagnosis and Treatment Options. Cureus.

[61] Borsini A., Zunszain P.A., Pfaff D., Christen Y. (2016). Advances in Stem Cells Biology: New Approaches to Understand Depression. Stem Cells in Neuroendocrinology.

[62] Vats A., Bielby R., Tolley N., Nerem R., Polak J. (2005). Stem Cells. Lancet.

[63] Falanga V. (2012). Stem Cells in Tissue Repair and Regeneration. J. Investig. Dermatol..

[64] Congdon C.C. (1957). Bone Marrow Transplantation in Animals Exposed to Whole-Body Radiation. J. Cell. Comp. Physiol..

[65] Kolios G., Moodley Y. (2013). Introduction to Stem Cells and Regenerative Medicine. Respiration.

[66] Mora C., Serzanti M., Consiglio A., Memo M., Dell’Era P. (2017). Clinical Potentials of Human Pluripotent Stem Cells. Cell Biol. Toxicol..

[67] Zhang Y.-T., He K.-J., Zhang J.-B., Ma Q.-H., Wang F., Liu C.-F. (2021). Advances in Intranasal Application of Stem Cells in the Treatment of Central Nervous System Diseases. Stem Cell Res. Ther..

[68] Villar-Gómez N., Ojeda-Hernandez D.D., López-Muguruza E., García-Flores S., Bonel-García N., Benito-Martín M.S., Selma-Calvo B., Canales-Aguirre A.A., Mateos-Díaz J.C., Montero-Escribano P. (2022). Nose-to-Brain: The Next Step for Stem Cell and Biomaterial Therapy in Neurological Disorders. Cells.

[69] Marcatili M., Sala C., Dakanalis A., Colmegna F., D’Agostino A., Gambini O., Dell’Osso B., Benatti B., Conti L., Clerici M. (2020). Human Induced Pluripotent Stem Cells Technology in Treatment Resistant Depression: Novel Strategies and Opportunities to Unravel Ketamine’s Fast-Acting Antidepressant Mechanisms. Ther. Adv. Psychopharmacol..

[70] Soliman M.A., Aboharb F., Zeltner N., Studer L. (2017). Pluripotent Stem Cells in Neuropsychiatric Disorders. Mol. Psychiatry.

[71] Correia-Melo F.S., Leal G.C., Carvalho M.S., Jesus-Nunes A.P., Ferreira C.B.N., Vieira F., Magnavita G., Vale L.A.S., Mello R.P., Nakahira C. (2018). Comparative Study of Esketamine and Racemic Ketamine in Treatment-Resistant Depression: Protocol for a Non-Inferiority Clinical Trial. Medicine.

[72] Licinio J., Wong M.-L. (2016). Serotonergic Neurons Derived from Induced Pluripotent Stem Cells (IPSCs): A New Pathway for Research on the Biology and Pharmacology of Major Depression. Mol. Psychiatry.

[73] Xu Z., Jiang H., Zhong P., Yan Z., Chen S., Feng J. (2016). Direct Conversion of Human Fibroblasts to Induced Serotonergic Neurons. Mol. Psychiatry.

[74] Vadodaria K.C., Mertens J., Paquola A., Bardy C., Li X., Jappelli R., Fung L., Marchetto M.C., Hamm M., Gorris M. (2016). Generation of Functional Human Serotonergic Neurons from Fibroblasts. Mol. Psychiatry.

[75] Uccelli A., Moretta L., Pistoia V. (2008). Mesenchymal Stem Cells in Health and Disease. Nat. Rev. Immunol..

[76] Ahmad F., Sachdeva P. (2022). A Consolidated Review on Stem Cell Therapy for Treatment and Management of Alzheimer’s Disease. Aging Med..

[77] Ding D.-C., Shyu W.-C., Lin S.-Z. (2011). Mesenchymal Stem Cells. Cell Transpl..

[78] Sun X., Hao H., Han Q., Song X., Liu J., Dong L., Han W., Mu Y. (2017). Human Umbilical Cord-Derived Mesenchymal Stem Cells Ameliorate Insulin Resistance by Suppressing NLRP3 Inflammasome-Mediated Inflammation in Type 2 Diabetes Rats. Stem Cell Res. Ther..

[79] Zhang Y., Wang X., Li Y., Liu R., Pan J., Tang X., Sun S., Liu J., Ma W. (2021). Human Umbilical Cord Mesenchymal Stem Cells Ameliorate Depression by Regulating Jmjd3 and Microglia Polarization in Myocardial Infarction Mice. Psychopharmacology.

[80] Mathews D.C., Henter I.D., Zarate C.A. (2012). Targeting the Glutamatergic System to Treat Major Depressive Disorder: Rationale and Progress to Date. Drugs.

[81] Mauri M.C., Ferrara A., Boscati L., Bravin S., Zamberlan F., Alecci M., Invernizzi G. (1998). Plasma and Platelet Amino Acid Concentrations in Patients Affected by Major Depression and under Fluvoxamine Treatment. Neuropsychobiology.

[82] Kim J.S., Schmid-Burgk W., Claus D., Kornhuber H.H. (1982). Increased Serum Glutamate in Depressed Patients. Arch. Psychiatr. Nervenkr..

[83] Mitani H., Shirayama Y., Yamada T., Maeda K., Ashby C.R., Kawahara R. (2006). Correlation between Plasma Levels of Glutamate, Alanine and Serine with Severity of Depression. Prog. Neuro-Psychopharmacol. Biol. Psychiatry.

[84] Frye M.A., Tsai G.E., Huggins T., Coyle J.T., Post R.M. (2007). Low Cerebrospinal Fluid Glutamate and Glycine in Refractory Affective Disorder. Biol. Psychiatry.

[85] Francis P.T., Poynton A., Lowe S.L., Najlerahim A., Bridges P.K., Bartlett J.R., Procter A.W., Bruton C.J., Bowen D.M. (1989). Brain Amino Acid Concentrations and Ca2+-Dependent Release in Intractable Depression Assessed Antemortem. Brain Res..

[86] Nowak G., Ordway G.A., Paul I.A. (1995). Alterations in the N-Methyl-d-Asparatate (NMDA) Receptor Complex in the Frontal Cortex of Suicide Victims. Brain Res..

[87] Holemans S., De Paermentier F., Horton R.W., Crompton M.R., Katona C.L.E., Maloteaux J.-M. (1993). NMDA Glutamatergic Receptors, Labelled with [3H]MK-801, in Brain Samples from Drug-Free Depressed Suicides. Brain Res..

[88] Hascup K.N., Hascup E.R., Stephens M.L., Glaser P.E., Yoshitake T., Mathé A.A., Gerhardt G.A., Kehr J. (2011). Resting Glutamate Levels and Rapid Glutamate Transients in the Prefrontal Cortex of the Flinders Sensitive Line Rat: A Genetic Rodent Model of Depression. Neuropsychopharmacology.

[89] Rajkowska G., Miguel-Hidalgo J.J., Wei J., Dilley G., Pittman S.D., Meltzer H.Y., Overholser J.C., Roth B.L., Stockmeier C.A. (1999). Morphometric Evidence for Neuronal and Glial Prefrontal Cell Pathology in Major Depression∗∗See Accompanying Editorial, in This Issue. Biol. Psychiatry.

[90] Öngür D., Drevets W.C., Price J.L. (1998). Glial Reduction in the Subgenual Prefrontal Cortex in Mood Disorders. Proc. Natl. Acad. Sci. USA.

[91] Yang C., Hu Y.-M., Zhou Z.-Q., Zhang G.-F., Yang J.-J. (2013). Acute Administration of Ketamine in Rats Increases Hippocampal BDNF and MTOR Levels during Forced Swimming Test. Upsala J. Med. Sci..

[92] Ghosh S., Sachdeva B., Sachdeva P., Chaudhary V., Rani G.M., Sinha J.K. (2022). Graphene Quantum Dots as a Potential Diagnostic and Therapeutic Tool for the Management of Alzheimer’s Disease. Carbon Lett..

[93] Shwartz A., Betzer O., Kronfeld N., Kazimirsky G., Cazacu S., Finniss S., Lee H.K., Motiei M., Dagan S.Y., Popovtzer R. (2017). Therapeutic Effect of Astroglia-like Mesenchymal Stem Cells Expressing Glutamate Transporter in a Genetic Rat Model of Depression. Theranostics.

[94] Friedman A., Frankel M., Flaumenhaft Y., Merenlender A., Pinhasov A., Feder Y., Taler M., Gil-Ad I., Abeles M., Yadid G. (2009). Programmed Acute Electrical Stimulation of Ventral Tegmental Area Alleviates Depressive-Like Behavior. Neuropsychopharmacology.

[95] Jia K.-K., Ding H., Yu H.-W., Dong T.-J., Pan Y., Kong L.-D. (2018). Huanglian-Wendan Decoction Inhibits NF- κ B/NLRP3 Inflammasome Activation in Liver and Brain of Rats Exposed to Chronic Unpredictable Mild Stress. Mediat. Inflamm..

[96] Huang X., Fei G., Liu W., Ding J., Wang Y., Wang H., Ji J., Wang X. (2020). Adipose-Derived Mesenchymal Stem Cells Protect against CMS-Induced Depression-like Behaviors in Mice via Regulating the Nrf2/HO-1 and TLR4/NF-ΚB Signaling Pathways. Acta Pharmacol. Sin..

[97] Kang H.S., Choi S.H., Kim B.S., Choi J.Y., Park G.-B., Kwon T.G., Chun S.Y. (2015). Advanced Properties of Urine Derived Stem Cells Compared to Adipose Tissue Derived Stem Cells in Terms of Cell Proliferation, Immune Modulation and Multi Differentiation. J. Korean Med. Sci..

[98] Jin M., Sheng W., Han L., He Q., Ji X., Liu K. (2018). Activation of BDNF-TrkB Signaling Pathway-Regulated Brain Inflammation in Pentylenetetrazole-Induced Seizures in Zebrafish. Fish Shellfish Immunol..

[99] Gao J., Xiong B., Zhang B., Li S., Huang N., Zhan G., Jiang R., Yang L., Wu Y., Miao L. (2018). Sulforaphane Alleviates Lipopolysaccharide-Induced Spatial Learning and Memory Dysfunction in Mice: The Role of BDNF-MTOR Signaling Pathway. Neuroscience.

[100] Jahromi M., Razavi S., Amirpour N., Khosravizadeh Z. (2016). Paroxetine Can Enhance Neurogenesis during Neurogenic Differentiation of Human Adipose-Derived Stem Cells. Avicenna J. Med. Biotechnol..

[101] Wang L.-J., Liu L.-P., Gu X.L., Wang M., Liu L.-M. (2018). Implantation of Adipose-Derived Stem Cells Cures the Optic Nerve Injury on Rats through Inhibiting the Expression of Inflammation Factors in the TLR4 Signaling Pathway. Eur. Rev. Med. Pharmacol. Sci..

[102] Putcha G.V., Le S., Frank S., Besirli C.G., Clark K., Chu B., Alix S., Youle R.J., LaMarche A., Maroney A.C. (2003). JNK-Mediated BIM Phosphorylation Potentiates BAX-Dependent Apoptosis. Neuron.

[103] Tfilin M., Sudai E., Merenlender A., Gispan I., Yadid G., Turgeman G. (2010). Mesenchymal Stem Cells Increase Hippocampal Neurogenesis and Counteract Depressive-like Behavior. Mol. Psychiatry.

[104] Kin K., Yasuhara T., Kameda M., Tomita Y., Umakoshi M., Kuwahara K., Kin I., Kidani N., Morimoto J., Okazaki M. (2020). Cell Encapsulation Enhances Antidepressant Effect of the Mesenchymal Stem Cells and Counteracts Depressive-like Behavior of Treatment-Resistant Depressed Rats. Mol. Psychiatry.

[105] Urbán N., Blomfield I.M., Guillemot F. (2019). Quiescence of Adult Mammalian Neural Stem Cells: A Highly Regulated Rest. Neuron.

[106] Kukekov V.G., Laywell E.D., Suslov O., Davies K., Scheffler B., Thomas L.B., O’Brien T.F., Kusakabe M., Steindler D.A. (1999). Multipotent Stem/Progenitor Cells with Similar Properties Arise from Two Neurogenic Regions of Adult Human Brain. Exp. Neurol..

[107] Alvarez-Buylla A., García-Verdugo J.M. (2002). Neurogenesis in Adult Subventricular Zone. J. Neurosci..

[108] Liu Z., Martin L.J. (2003). Olfactory Bulb Core Is a Rich Source of Neural Progenitor and Stem Cells in Adult Rodent and Human. J. Comp. Neurol..

[109] Kempermann G. (2002). Regulation of Adult Hippocampal Neurogenesis—Implications for Novel Theories of Major Depression 1: Regulation of Adult Hippocampal Neurogenesis. Bipolar Disord..

[110] Mansoor A.K., Thomas S., Sinha J.K., Alladi P.A., Ravi V., Raju T.R. (2012). Olfactory tract transection reveals robust tissue-level plasticity by cellular numbers and neurotrophic factor expression in olfactory bulb. Indian J. Exp. Biol..

[111] Feldmann R.E., Mattern R. (2006). The Human Brain and Its Neural Stem Cells Postmortem: From Dead Brains to Live Therapy. Int. J. Leg. Med..

[112] Lindvall O., Kokaia Z. (2004). Recovery and Rehabilitation in Stroke: Stem Cells. Stroke.

[113] Sachdeva P., Ghosh S., Ghosh S., Han S., Banerjee J., Bhaskar R., Sinha J.K. (2022). Childhood Obesity: A Potential Key Factor in the Development of Glioblastoma Multiforme. Life.

[114] Ghosh S., Manchala S., Raghunath M., Sharma G., Singh A.K., Sinha J.K. (2021). Role of Phytomolecules in the Treatment of Obesity: Targets, Mechanisms and Limitations. Curr. Top. Med. Chem..

[115] Goldman S. (2005). Stem and Progenitor Cell–Based Therapy of the Human Central Nervous System. Nat. Biotechnol..

[116] Lipska B.K. (2004). Using Animal Models to Test a Neurodevelopmental Hypothesis of Schizophrenia. J. Psychiatry Neurosci..

[117] Feldmann R.E., Sawa A., Seidler G.H. (2007). Causality of Stem Cell Based Neurogenesis and Depression—To Be or Not to Be, Is That the Question?. J. Psychiatr. Res..

[118] Zhao C. (2006). Distinct Morphological Stages of Dentate Granule Neuron Maturation in the Adult Mouse Hippocampus. J. Neurosci..

